# Meniscal ramp lesions: an illustrated review

**DOI:** 10.1186/s13244-021-01080-9

**Published:** 2021-09-25

**Authors:** Atul K. Taneja, Frederico C. Miranda, Laercio A. Rosemberg, Durval C. B. Santos

**Affiliations:** 1 Musculoskeletal Radiology Division, Imaging Department, Hospital Israelita Albert Einstein, São Paulo, SP Brazil; 2 Departamento de Imagem – Hospital Israelita Albert Einstein, Av. Albert Einstein, 627, Morumbi, São Paulo, SP CEP 05652-900 Brazil

**Keywords:** Ramp lesion, Meniscus, MRI, Arthroscopy, ACL tear

## Abstract

The purpose of this review is to describe the anatomy and lesions affecting the peripheral portion of posterior horn of medial menisci (ramp lesions), along with illustrations and MRI cases. We will correlate imaging features with arthroscopic classification of ramp lesions. Also, postoperative and chronic changes related to meniscocapsular tears will be presented, as well as biomechanical consequences and treatment approach.

## Key points

- Peripheral injuries affecting posterior horn of medial meniscus in the setting of ACL tears are called ramp lesions.
- Ramp lesions include peripheral tears, meniscocapsular separation and meniscotibial ligament injuries.
- Some classifications have addressed meniscal ramp lesions, with good reproducibility by MRI.
- Surgical exploration of posteromedial compartment of the knee is needed.

## Background

Meniscal ramp lesions consist in longitudinal vertical and/or oblique peripheral tears affecting the posterior horn of medial meniscus that may lead to meniscocapsular or meniscotibial disruption, in the setting of an ACL tear [1].

The coexistence of an ACL tear and other capsular and ligament injuries has been extensively described [2]. Acute ACL tear is associated with meniscal injuries in more than 50% of subjects, and in more than 80% of chronic ACL tear cases. Medial meniscus is firmly attached to the tibia and femur, acting as a knee stabilizer, and preventing anterior translation, especially in the ACL-deficient knee, thus being especially susceptible to injuries [3].

Ramp lesions have important biomechanical consequences, and they occur much more frequently than thought. Ramp lesions remain significantly underdiagnosed and therefore are not promptly surgically repaired in standard knee arthroscopies, since it relies on anterior portals, limiting a full assessment of the posterior horn and attachment of the medial meniscus [4].

Recent data and definition of risk factors bring an appropriate index of suspicion, identification, and adequate therapeutic planning for ramp lesions [5]. A systematic approach using MRI, and especially, arthroscopic exploration of the posteromedial compartment of the knee using a specific trans-notch approach is needed to clearly assess a meniscal ramp lesion [6].

Since this type of injury is often missed, both during MRI reading and due to its "blind" point of arthroscopic vision, it is crucial to make an accurate preoperative diagnosis. The aim of this article is to educate in an illustrative manner the recent literature findings of meniscal ramp lesions, including its anatomical, biomechanical and diagnostic features.

## Definition and incidence

Meniscal ramp lesions can be defined as longitudinal vertical and/or oblique peripheral tears affecting posterior horn of medial meniscus, in a mediolateral direction of less than 2.0 cm, that may lead to meniscocapsular or meniscotibial disruption [1]. Medial meniscal ramp lesions are reported to be present in 15% [7], 20% [1] to 42% [8] of patients undergoing anterior cruciate ligament (ACL) reconstruction.

## Risk factors

A study by Kim et al. [9] highlights that significant risk factors for ramp lesions include bone contusion at the posterior medial tibial plateau, chronic ACL injury, steeper medial tibial and meniscal slope, gradual lateral tibial slope, and varus knee > 3°.

In a recently published systematic review and meta-analyses by Kunze et al. [10], significant associations between male sex, age < 30 years, posteromedial tibial edema on magnetic resonance imaging (MRI), concomitant lateral meniscal tears, complete ACL tears, injury chronicity, and the presence of ramp lesions were found. On the other hand, contact injury and revision of ACL reconstruction were not significantly associated with the presence of ramp lesions.

Moreover, increased medial meniscal slope was identified to be an independent anatomic risk factor of concomitant ramp lesions in noncontact ACL injuries [11], and the presence of posterior medial tibial plateau edema demonstrated significantly greater odds for ramp lesions compared with meniscal body tears [7].

## Posterior meniscal anatomy

**Fig. 1:**
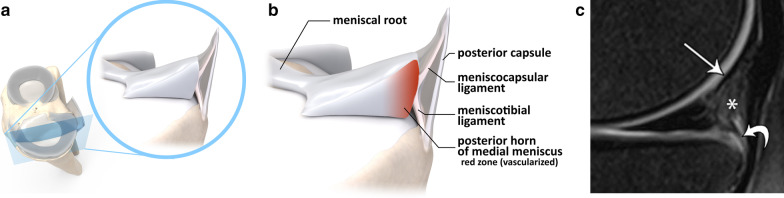
Posterior medial meniscus anatomy, with its corresponding structures. In (**a**), an illustrated open view of the medial compartment of the knee, in (**b**), a zoomed view of the posteromedial capsular-meniscal unit, and in (**c**), the corresponding MRI appearance on sagittal PD-weighted fat suppressed: meniscocapsular ligament (thin arrow), meniscotibial ligament (curved arrow) and posterior capsular attachment (star)

The posterior capsular junction of medial meniscus is composed by the meniscocapsular ligament superiorly and the meniscotibial ligament inferiorly [4], as detailed in Fig. 1. Together with the posterior horn of the medial meniscus, they stabilize the knee against anterior tibial translation and posteromedial rotation.

Ramp lesions occur in the peripheral zone of the medial meniscus (red-red area), where meniscocapsular and meniscotibial structures attach to the posterior horn.

Ramp lesions can occur during traumatic acute ACL injury or increased tibial translation in chronic ACL-deficient knee.

## Imaging diagnosis

Ramp lesions can be detected in routine MRI imaging of the knee, although dedicated flexed-knee and/or 3D sequences for ligament and meniscal injuries have shown to be promising [12, 13]. MRI studies present variable sensitivity and specificity for the detection of meniscal ramp lesions, such as

- Arner et al. [14] reported sensitivity of 53–84% and specificity of 92–98%;
- DePhillipo et al. [1] reported a sensitivity of 48%;
- Koo et al.’s [15] systematic review and meta-analyses resulted in sensitivity of 71% and specificity of 94.

Sensitivity of 3.0-T MRI (83.3%) was superior to 1.5-T MRI (67.6%), according to Hatayama et al. [16]. MRI exams of the knee in our institution follow a departmental protocol in supine position and leg extension, using a phased array dedicated coil, on 1.5­T or 3.0-T scanners. The majority of the exams use include: sagittal T1-weighted, sagittal, axial and coronal proton-density (PD) fat-suppressed or T2-weighted fat-suppressed sequences.

Irregularity at the posterior margin and complete fluid filling were the most sensitive findings for detecting ramp lesion on MRI, according to Yeo et al. [17] and Laurens et al. [18]. Consecutive sagittal images serve to determine which regions of the meniscocapsular junction and posterior horn are torn. Axial images help to assess mediolateral dimensions of the same lesions.

### MRI features

1. Thin fluid signal completely interposed between the posterior horn of the medial meniscus and the posteromedial capsule.
2. Longitudinal vertical and/or oblique tear affecting the peripheral zone of the posterior horn of the medial meniscus.
3. Irregularities involving the posterior margin of medial meniscus, with focal discontinuity or step-like deformity, and involving capsular attachments.
4. Soft tissue edema between meniscus and collateral ligament.
5. Bone bruise of posteromedial tibia from pivot shift countercoup injury in medial compartment, and anterior translation of medial plateau in relation to femoral condyle.
6. Signs of concurrent ACL injury [4].

## Classifications

Few classifications have addressed meniscal ramp lesions, as follows:

- Seil et al. [19] approached the mediolateral extent of tears, degree of capsular attachment injury, and adherent (self-heal) vs. dehiscent (repair).
- Thaunat et al. [20] approached the tear pattern, direction, thickness (partial vs. full), and associated meniscocapsular disruption, peripheral zone, or meniscotibial ligament lesion and instability.
- Greif et al. [4] in an extended Thaunat classification version integrate the recent knowledge from cadaveric studies showing that meniscocapsular and meniscotibial ligaments merge in their posterior horn meniscal attachment.

Table 1 brings a comparison between Thaunat’s and Grief’s classifications. In summary: ramp lesion type 1 is an isolated posterior superior meniscocapsular tear (Figs. 2 and 3); type 2 is a partial posterior superior tear (Figs. 4 and 5); type 3 is partial posterior inferior tear (Figs. 6 and 7) or meniscotibial ligament tear (Figs. 8 and 9); type 4 is a complete posterior peripheral tear (Figs. 10 and 11) or complete meniscocapsular junction tear (Figs. 12 and 13); and type 5 is a posterior horn double tear (Figs. 14 and 15).

**Table 1:** Correlation between Thaunat’s and Greif’s classifications for meniscal ramp lesions, with corresponding illustrations

Ramp lesion classifications		
Thaunat et al	Greif et al	Illustration
**Type 1**: meniscocapsular tear	**Type 1**: meniscocapsular ligament tear	
**Type 2**: partial superior tear	**Type 2**: partial superior peripheral posterior meniscal horn tear	
**Type 3**: partial inferior tear	**Type 3A**: partial inferior peripheral posterior horn meniscal tear	
	**Type 3B**: meniscotibial ligament tear	
**Type 4**: complete tear	**Type 4A**: complete peripheral posterior horn meniscal tear	
	**Type 4B**: complete meniscocapsular junction tear	
**Type 5**: double tear	**Type 5**: peripheral posterior horn meniscal double tear

**Fig. 2:**
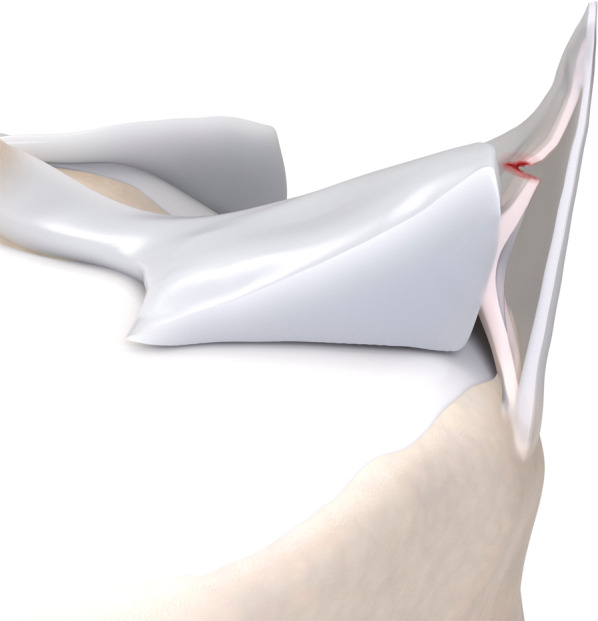
Type 1 ramp lesion illustration, defined as meniscocapsular ligament tear

**Fig. 3:**
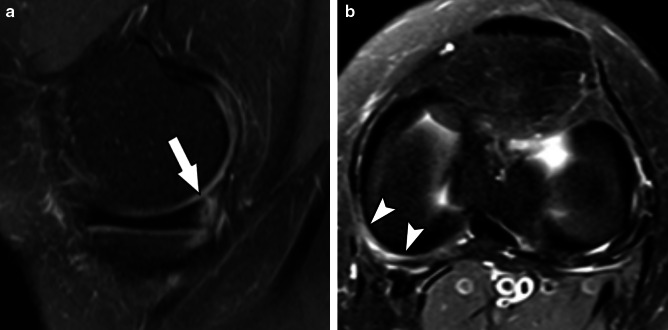
Type 1 meniscal ramp lesion MRI Case. Sagittal (**a**) and axial T2-weighted fat-suppressed images show meniscocapsular tear (arrow) with tibial bone bruise, and the extension of meniscocapsular junction edema (arrowheads)

**Fig. 4:**
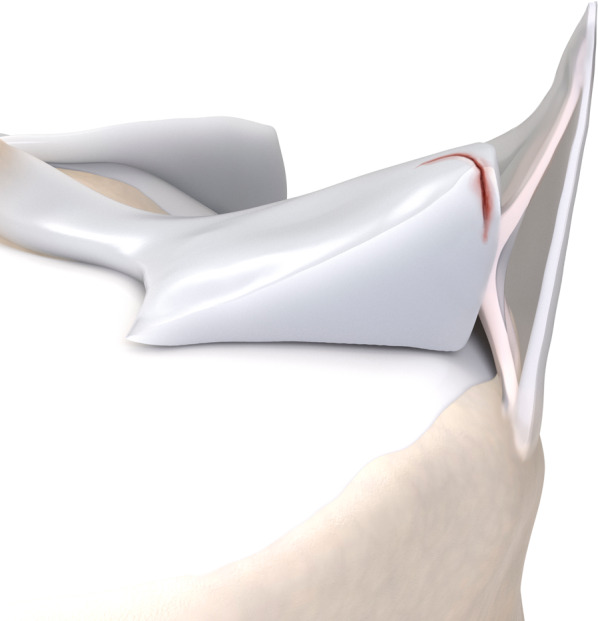
Type 2 meniscal ramp lesion illustration, defined as partial superior peripheral posterior meniscal horn tear

**Fig. 5:**
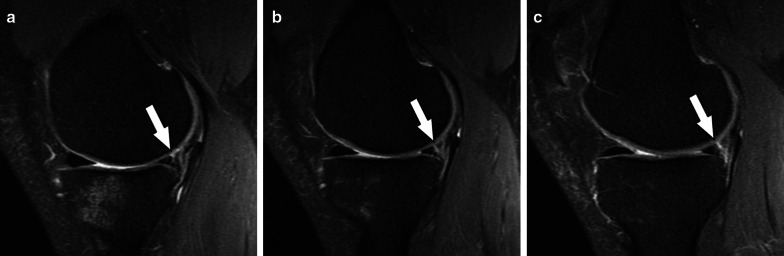
Type 2 meniscal ramp lesion MRI Case. Sequential sagittal T2-weighted fat-suppressed MR images show peripheral partial tear of posterior horn of medial meniscus (arrows) affecting the femoral articular surface, along with meniscocapsular ligament tear

**Fig. 6:**
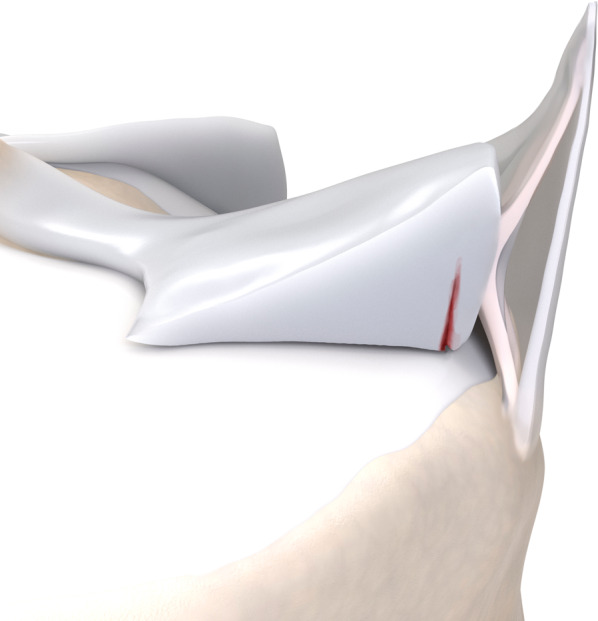
Type 3A meniscal ramp lesion illustration, defined as partial inferior peripheral posterior horn meniscal tear

**Fig. 7:**
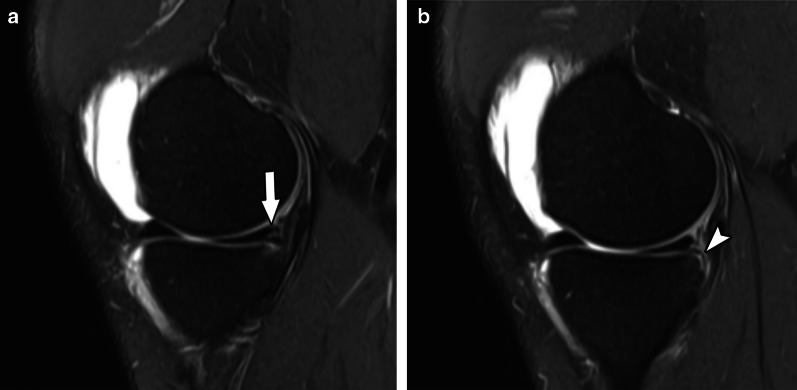
Type 3A meniscal ramp lesion MRI Case. Sagittal T2-weighted fat-suppressed images show peripheral vertical partial tear (arrow in **a**) extending to the tibial surface of the posterior horn of medial meniscus, with intact meniscotibial ligament (arrowhead in **b**)

**Fig. 8:**
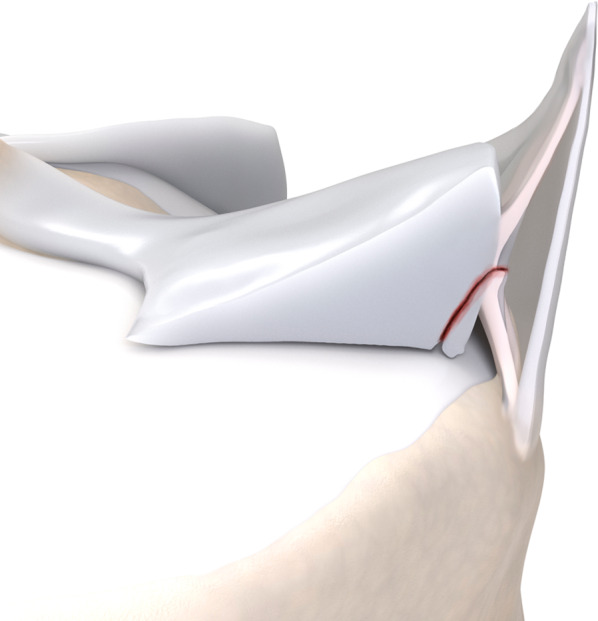
Type 3B meniscal ramp lesion illustration, defined as a partial inferior tear affecting the meniscotibial ligament

**Fig. 9:**
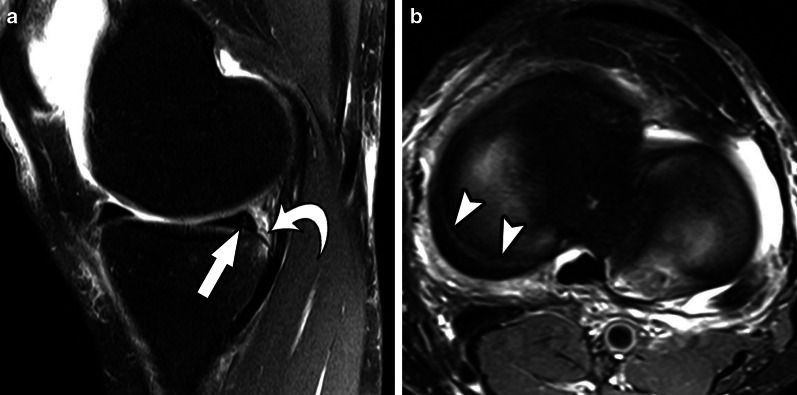
Type 3B meniscal ramp lesion MRI Case. Sagittal (**a**) and axial (**b**) T2-weighetd fat-suppressed images show peripheral partial vertical tear affecting the tibial surface of the posterior horn of medial meniscus (arrow), along with meniscotibial ligament tear (curved arrow) and bone bruise; extension of vertical tear is seen in (**b**) (arrowheads)

**Fig. 10:**
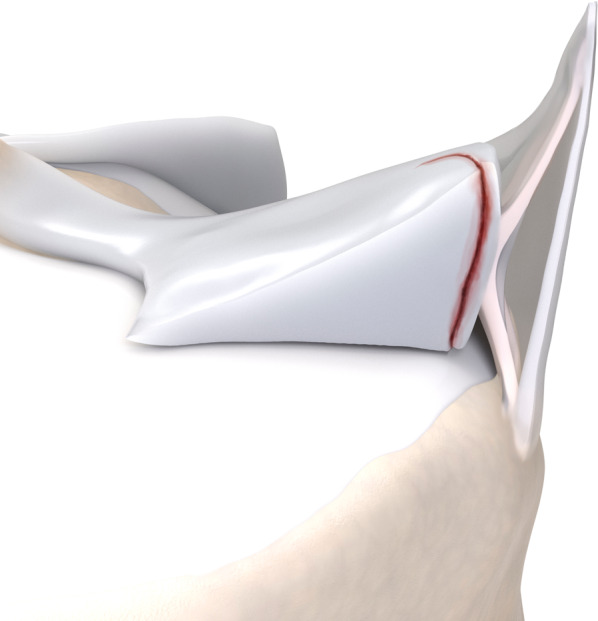
Type 4A meniscal ramp lesion illustration, defined as complete peripheral posterior horn meniscal tear

**Fig. 11:**
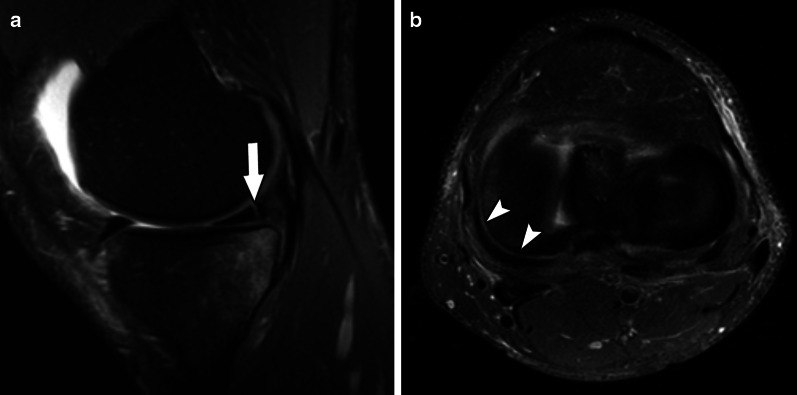
Type 4A meniscal ramp lesion MRI Case. Sagittal (**a**) and axial (**b**) T2-weighted fat-suppressed MR image shows a type IV ramp lesion, with tear extending from femoral to tibial articular surfaces (arrow); tear extension is seen in (**b**) (arrowheads)

**Fig. 12:**
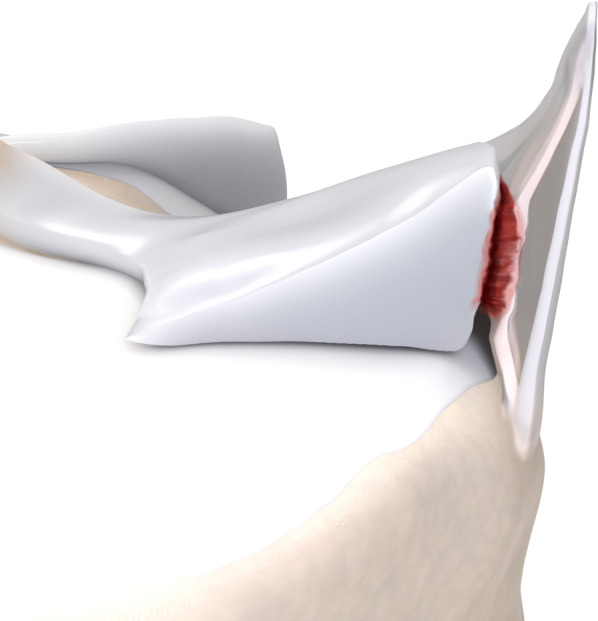
Type 4B meniscal ramp lesion illustration, defined as complete meniscocapsular junction tear

**Fig. 13:**
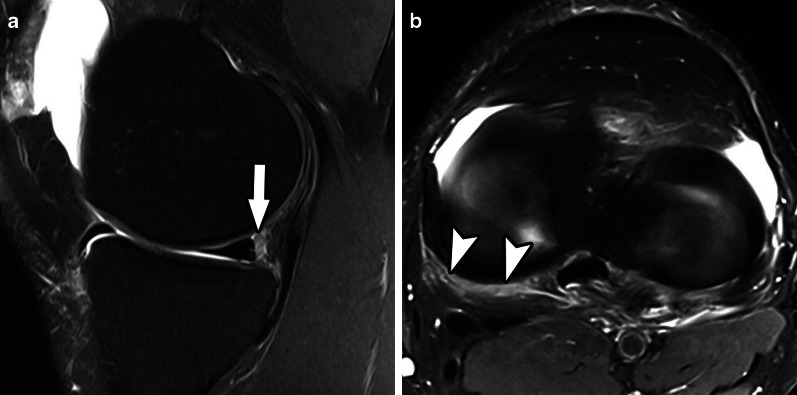
Type 4B meniscal ramp lesion MRI case. Sagittal (**a**) T2-weighted fat-suppressed MR image shows a type IV ramp lesion, with a complete meniscocapsular disruption of the posterior horn of medial meniscus. Axial (**b**) image shows a cross-sectional view of the extensive meniscocapsular disruption

**Fig. 14:**
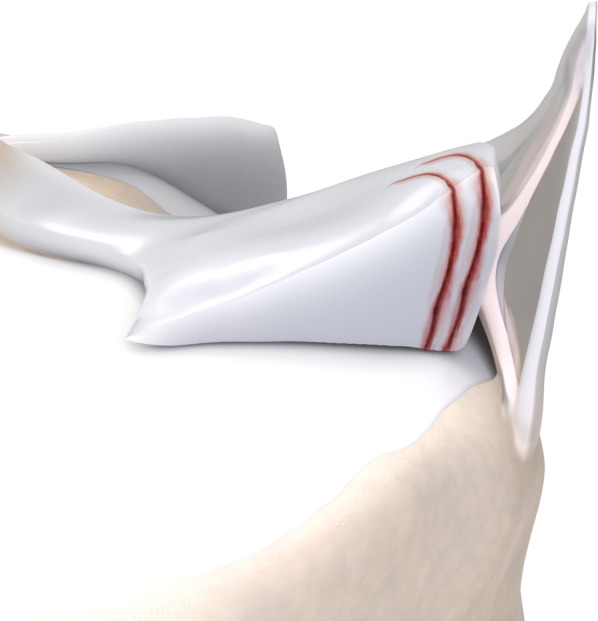
Type 5 meniscal ramp lesion illustration, defined as peripheral posterior horn meniscal double tear

**Fig. 15:**
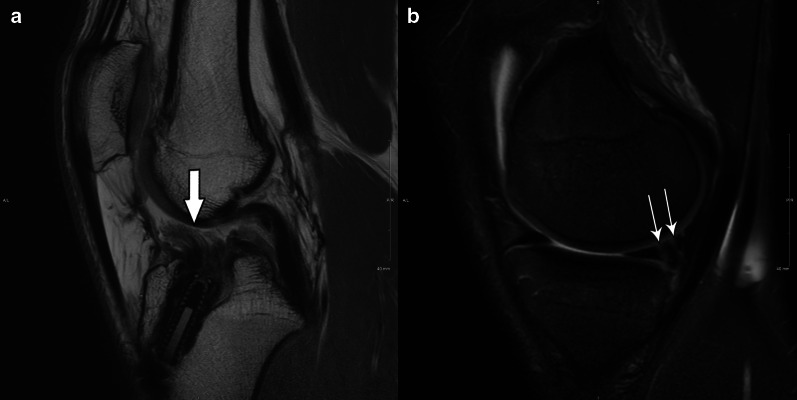
Type 5 meniscal ramp lesion MRI case. Sagittal PD-weighted fat-suppressed MR image (**a**, left) shows a postoperative ACL graft tear (arrow), and sagittal T2-weighted fat-suppressed (**b**, right) shows a double vertical peripheral tear through the posterior horn of medial meniscus (thin arrows). *Case courtesy of Dr. Diego Lemos (USA)

Ramp lesions type 1 and 2 are usually considered stable, and types 3 to 5 as unstable [20]. MRI has shown a good reproducibility of the arthroscopy stability classification for meniscal ramp lesions, when applied by trained musculoskeletal radiologists [21].

## Treatment approach and postoperative appearance

**Fig. 16:**
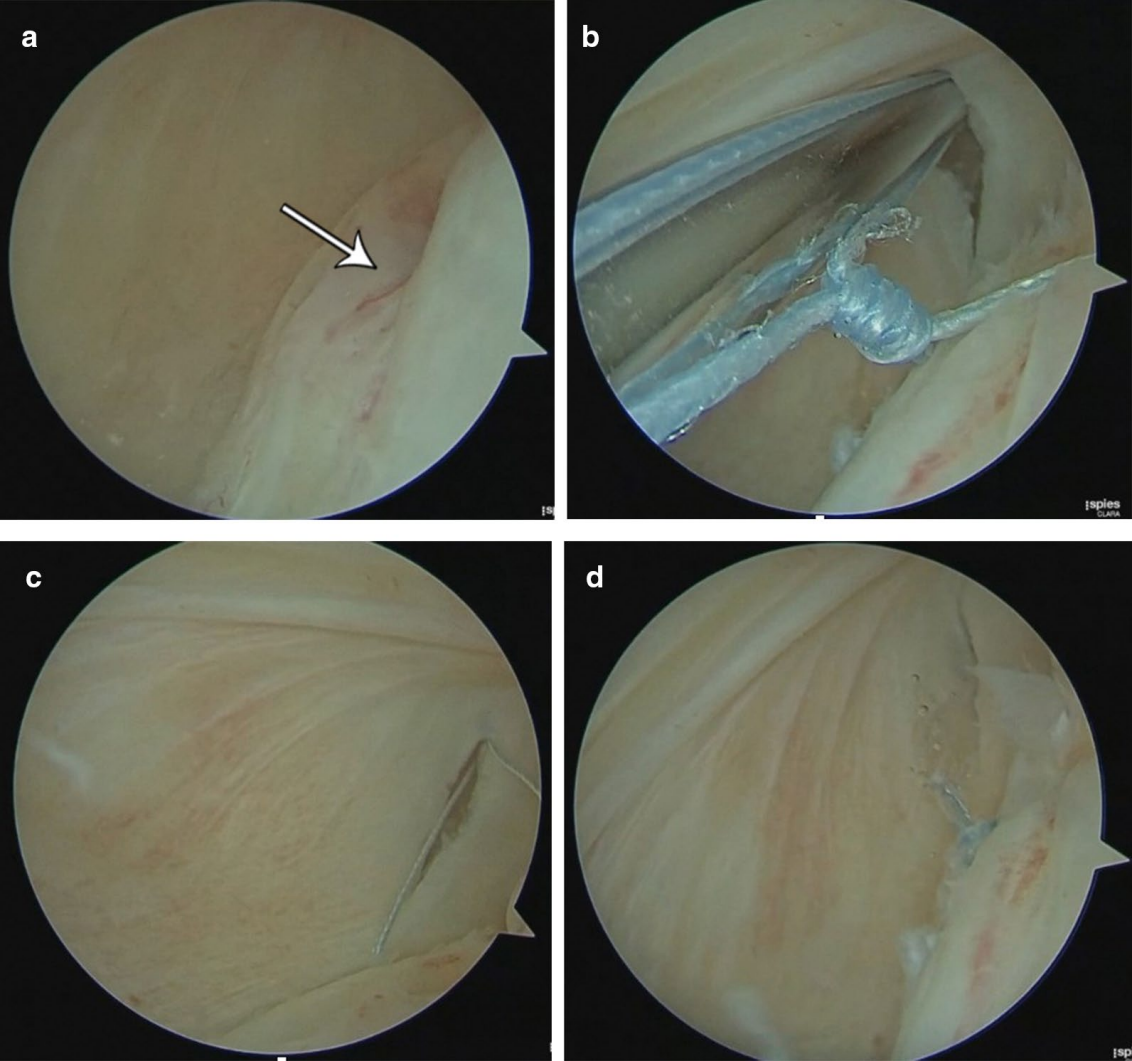
Arthroscopic images presents intra-articular diagnosis of a Type 4 ramp lesion through a posteromedial portal (**a**). An all-inside meniscal repair was performed (**b** and **c**). Final suture aspect is seen in (**d**). *Case courtesy of Dr. Luis. E. P. Tirico (Brazil)

**Fig. 17:**
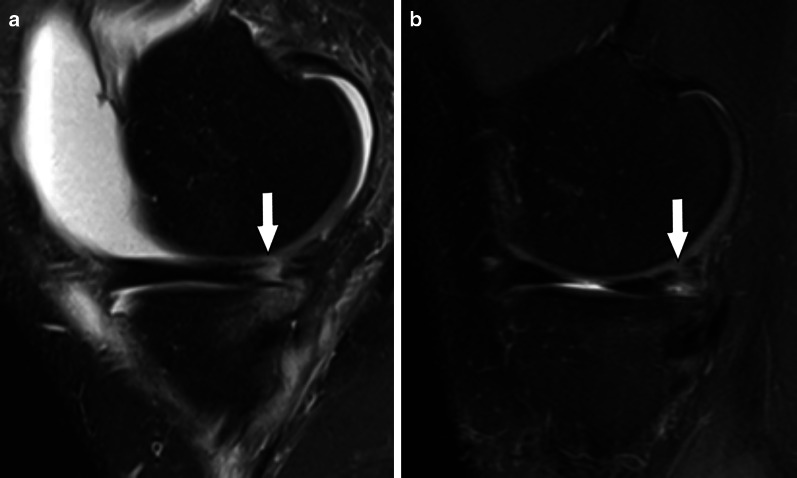
(**a**) Sagittal fat-suppressed MR image shows a type 4 acute ramp lesion (arrow) and in (**b**) the postoperative appearance after 4 months of surgery (arrow)

Since MRI demonstrates moderate accuracy specificity for diagnosing ramp lesion, arthroscopic assessment is recommended to asses peripheral meniscal tears, even if the MRI is not clear [15]; Figs. 16 and 17.

## Differential diagnosis

**Fig. 18:**
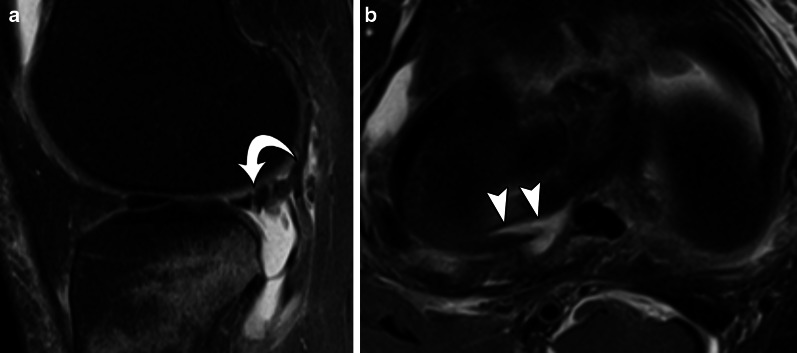
Sagittal T2-weighted fat-suppressed MR image (**a**) shows a zip lesion, with a rip between *Wrisberg* ligament (curved arrow) and posterior horn of lateral meniscus. Axial T2-weighetd fat-suppressed MR image (**b**) shows the lesion in a cross-sectional view (arrowheads)

**Fig. 19:**
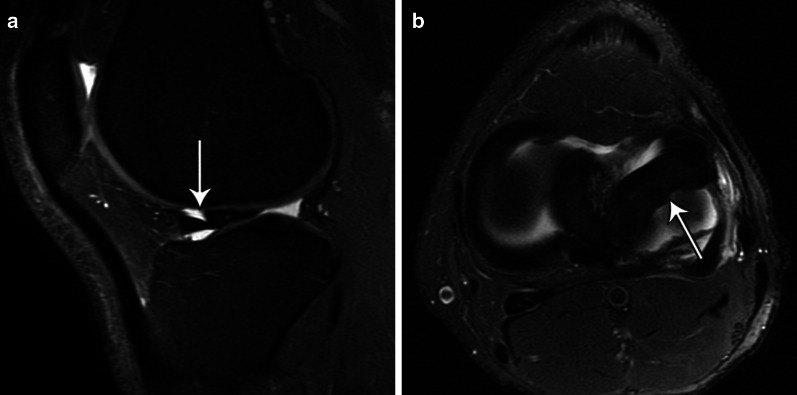
Sagittal T2-weighted fat-suppressed MR image (**a**) shows a double delta sign (thin arrow), typical for flipped meniscus. Axial image (**b**) shows anterior dislocation of the posterior horn of lateral meniscus (thin arrow)

**Fig. 20:**
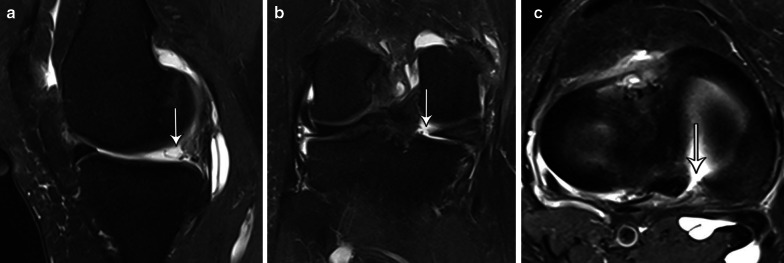
Sagittal (**a**), coronal (**b**) and axial (**c**) T2-weighetd fat-suppressed MR images show “ghost sign” on posterior medial meniscal root attachment (thin arrows). Full-thickness tear of the posterior medial meniscus root is clearly demonstrated in (**b**) and (**c**)

- Zip Lesion (“Wrisberg rip”, “Zipper tear”) are longitudinal vertical and/or oblique meniscal tears, located in the junction of the Wrisberg meniscofemoral ligament and the posterior horn of the lateral meniscus and are also associated with anterior cruciate ligament tears (Fig. 18). The tear must extend more than 1.4 cm in a mediolateral direction, from the lateral edge of the posterior cruciate ligament, differentiating from a not so uncommon pitfall, the “Wrisberg pseudotear” [22].
- Flipped meniscus refers to a subtype of a “bucket handle” tear with a displaced anteriorly meniscal fragment adjacent to the anterior horn of the meniscus that seems enlarged with an irregular contour (Fig. 19).
- Meniscal root tear are radial tears or bony avulsions located within 1.0 cm from the meniscal attachment to the central tibial plateau (Fig. 20) that may have serious biomechanical consequences, including meniscal extrusion, secondary osteoarthritis and subchondral insufficiency.

## Biomechanical consequences

Meniscal ramp lesions are related to increased anterior translation, rotational laxity, and excessive knee motion, leading to increased biomechanical instability of the knee [4] when compared to subjects with isolated ACL tear.

Failure to appropriately identify and repair medial meniscal ramp lesions at the time of anterior cruciate ligament reconstruction may result in increased anterior tibial translation and internal rotation, increasing the risk for graft failure [10].

Either in Thaunat’s or Greif’s classification, types 1 and 2 are considered stable, while types 3, 4 and 5 are considered unstable. If left untreated, ramp lesions may contribute to create anteroposterior instability related to an ACL–reconstructed knee, leading to graft or meniscal repair failure [4].

A recent study by Guimaraes et al. [23] showed that knees with meniscal ramp lesions demonstrated accelerated degeneration of cartilage composition in the medial knee compartment over 2 years, evaluated by T1ρ and T2 mapping.

## Conclusion

MRI findings related to posterior meniscal injuries, especially “ramp” (medial meniscus) and “zip” (lateral meniscus) lesions, include peripheral meniscal tears, meniscocapsular separation and/or meniscotibial ligament tear. Such meniscal lesions occur usually in the setting of anterior cruciate ligament ruptures, and MRI adds relevant imaging information enabling proper surgical planning.

## Data Availability

Data sharing is not applicable to this article as no datasets were generated or analyzed during the current study.

## Abbreviations

ACL: Anterior cruciate ligament
MRI: Magnetic Resonance Imaging
